# Ultrasensitive Electrical Detection of Hemagglutinin for Point-of-Care Detection of Influenza Virus Based on a CMP-NANA Probe and Top-Down Processed Silicon Nanowire Field-Effect Transistors

**DOI:** 10.3390/s19204502

**Published:** 2019-10-17

**Authors:** Mihee Uhm, Jin-Moo Lee, Jieun Lee, Jung Han Lee, Sungju Choi, Byung-Gook Park, Dong Myong Kim, Sung-Jin Choi, Hyun-Sun Mo, Yong-Joo Jeong, Dae Hwan Kim

**Affiliations:** 1 School of Electrical Engineering, Kookmin University, Seoul 02707, Korea; iljss88@naver.com (M.U.); taiji11jieun@kookmin.ac.kr (J.L.); sungjuchoi@kookmin.ac.kr (S.C.); dmkim@kookmin.ac.kr (D.M.K.); sjchoiee@kookmin.ac.kr (S.-J.C.); tyche@kookmin.ac.kr (H.-S.M.); 2 School of Applied Chemistry, Kookmin University, Seoul 02707, Korea; elzem@kookmin.ac.kr; 3 School of Electrical and Computer Engineering, University of Seoul, Seoul 02504, Korea; kusa159@snu.ac.kr (J.H.L.); bgpark@snu.ac.kr (B.-G.P.)

**Keywords:** influenza virus, HA1, hemagglutinin, silicon nanowire biosensor, label-free, point-of-care

## Abstract

Rather than the internal genome nucleic acids, the biomolecules on the surface of the influenza virus itself should be detected for a more exact and rapid point-of-care yes/no decision for influenza virus-induced infectious diseases. This work demonstrates the ultrasensitive electrical detection of the HA1 domain of hemagglutinin (HA), a representative viral surface protein of the influenza virus, using the top-down complementary metal oxide semiconductor (CMOS) processed silicon nanowire (SiNW) field-effect transistor (FET) configuration. Cytidine-5′-monophospho-N-acetylneuraminic acid (CMP-NANA) was employed as a probe that specifically binds both to the aldehyde self-aligned monolayer on the SiNWs and to HA1 simultaneously. CMP-NANA was serially combined with two kinds of linkers, namely 3-aminopropyltriethoxysilane and glutaraldehyde. The surface functionalization used was verified using the purification of glutathione S-transferase-tagged HA1, contact angle measurement, enzyme-linked immunosorbent assay test, and isoelectric focusing analysis. The proposed functionalized SiNW FET showed high sensitivities of the threshold voltage shift (ΔV_T_) ~51 mV/pH and the ΔV_T_ = 112 mV (63 mV/decade) with an ultralow detectable range of 1 fM of target protein HA1.

## 1. Introduction

Swine flu (H1N1), which is swan-originated and occurred in Mexico in 2009, has caused more than 17,000 cumulative deaths worldwide [1]. Adequate and convenient diagnostic tools for the rapid and accurate detection of the influenza virus from infected carriers are indispensable in controlling the wide spread of the virus at an early stage of its outbreak. However, the previously established and widely used methods for virus detection, such as enzyme-linked immunosorbent assay (ELISA) [2,3,4,5,6], polymerase chain reaction (PCR) [6,7,8,9], and fluorescence-based methods [6,10,11], immunoblotting [12], immunosensor-based methods [13], interferometry [14], fluoro-immunoassay [15,16], surface plasmon resonance-based methods [17], immunochromatography [18], gold-star optical methods [6], GOLD SIGN FLU [19], and rapid diagnostic tests with digital readout system [20], have been unable to satisfy these explosive demands because most of them cannot achieve a rapid (<10 s) detection and often require a relatively high level of sample manipulation that is inconvenient for infectious materials. Highly skilled personnel and expensive laboratory instruments are also required to perform these methods.

To overcome these limitations and challenging issues encountered by the previous methods, a few approaches for point-of-care (POC) tests that perform rapid and label-free electrical sensing of ions or biomolecules have been demonstrated. These tests are based on ion-sensitive field-effect transistors (FETs) configurations with silicon nanowires (SiNWs), carbon nanotubes, AlGaN/GaN heterostructure, and ZnO nanotubes [21,22,23,24,25,26,27,28]. As is well known, FET-based electrical sensing biomolecules (including viruses) achieves faster detection than other methods [6]. Indeed, FET-based biomolecular sensors detect biomolecules within 10 s [26,27,28]. This detection time is two orders of magnitude smaller than the sensing time of RT-qPCR, the gold standard of biomolecular detection [29]. Moreover, the ion-sensitive FET (ISFET) array has been further industrialized as the extended-gate ISFET (EG-ISFET) [30,31,32].

**Figure 1:**
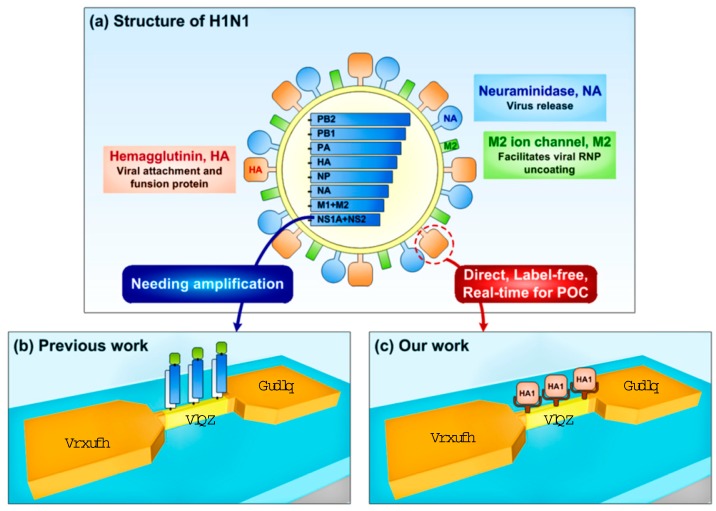
(**a**) Illustration of influenza virus structure. (**b**,**c**) Two schemes of electrical sensing of influenza virus with SiNWs; the detection of (**b**) specific nucleic acids sequences inside of influenza virus and (**b**) HA1 proteins on the surface of influenza virus itself that is involved in the initiation of infection.

However, the previous schemes of electrically sensing the influenza virus have a critical weakness. Figure 1a schematically illustrates the influenza virus structure. Figure 1b,c show the two possible schemes of electrically sensing the influenza virus with the SiNWs. Note that the previous approaches tried to detect specific nucleic acid sequences (Figure 1b) rather than the virus particles themselves (Figure 1c) [6]. In fact, the nucleic acids exist not on the surface, but inside the virus particles as seen in Figure 1a. The electrical detection of nucleic acids as an indicator of a viral infection does not play a critical role as a potential POC platform to help a yes/no decision for infectious diseases in a doctor’s clinic or at an airport because to extract nucleic acids as a virus infection marker, the specialized personnel needs to amplify specific nucleic acid sequences from the suspected patients’ bodies. Furthermore, the influenza virus can live outside of the host cell as a virion, and it infects the host while floating in the air. It can survive even in objects touched or used by the infected patients. Although the survival time covers various ranges according to its surficial condition, it can nominally survive for approximately 5 min in the skin, 15 min in a dry paper tissue, and 1 or 2 days on a plastic surface. The virus can survive much longer if it exists in mucus [33,34]. Therefore, the biomolecules on the surface of the influenza virus itself, rather than the internal genome nucleic acids, should be paid very careful attention to determine a rapid and accurate method that would detect the influenza virus.

The influenza virus generally drifts as a virion through the air until it infects a host. During the infection process, it secretes a viral surface protein called hemagglutinin (HA) (Figure 1a). The influenza HA protein determines the species that can be infected by a strain, and the binding location of the strain in the human respiratory tract [35]. In the strains that are easily transmitted among people, the HA proteins bind to receptors in the upper part of the respiratory tract, such as the nose, throat, and mouth. Thus, the detection of HAs involved in the initiation of infection will most efficiently and accurately recognize the existence of viruses in the human body. HA is an antigenic homotrimeric integral membrane glycoprotein. The HA monomer consists of a topped large HA1 globule and a long, helical chain anchored in the membrane by HA2 domains [36,37,38,39]. The HA1 domain contains sialic acid binding sites, and is well known to be essential for viral infection of host cells.

Inspired by these developments, we herein report the electrical and ultrahigh sensitive detection of HA1 using SiNW FETs fabricated by a complementary metal oxide semiconductor (CMOS)-compatible process. This is the first demonstration of electrical detection of the HA1 domain in HA, the viral surface protein of the influenza virus, using a top-down SiNW-based approach (Figure 1c). The surface functionalization with the effective probe and the intermediate linker, such as cytidine-5′-monophospho-N-acetylneuraminic acid (CMP-NANA) and glutaraldehyde (GA), and the high surface-to-volume ratio of the SiNWs revealed an ultrahigh sensitive detection (1 fM) of HA1 with a threshold voltage shift of 112 mV (63 mV/dec). 

## 2. Experimental Section

### 2.1. Purification of the GST-Tagged HA1 (HA1-GST)

The HA1 domain of the influenza virus was prepared as a target biomolecule through the purification of glutathione S-transferase (GST)-tagged HA1 (i.e., HA1-GST). The genomic RNA of A/Korea/01/2009(H1N1) was provided by Korea Centers for Disease Control and Prevention (KCDC). The cDNAs were obtained by reverse transcription using a high-capacity RNA-to-cDNA kit (Applied Biosystems, USA). The HA1 gene was amplified by PCR using G-Taq DNA polymerase (Cosmogenetech, Korea) with the *Eco*RI forward primer (5′-GTGGTGGAATTCGACACATTATGTATAGGTTATCATGCG-3′) and the *Xho*I reverse primer (5′-CATATTCTCGAGTCATCTAGATTGAATAGACGG-3′). The PCR product was gel-purified, digested with *Eco*RI/*Xho*I, and ligated with a similarly digested pGEX-4T-1 plasmid to make the expression vector, pGEX-4T-1/HA1. The HA1-GST protein expression plasmid, pGEX-4T-1/HA1, was transformed into *E. coli* Rosetta 2(DE3)^TM^ competent cells. Ten liters of LB broth supplemented with ampicillin (50 μg/mL) was inoculated with the saturated pGEX-4T-1/HA1/Rosetta 2(DE3)^TM^ culture (1/200 dilution) and grown at 37 °C until A_600_ = 0.8. The HA1-GST protein expression was induced by the addition of IPTG (0.5 mM). The cultures were then further incubated at 37 °C for 4 h. After cooling to 4 °C, the cells were harvested by centrifugation and resuspended in a lysis buffer (1× phosphate-buffered saline (PBS), 0.3 M NaCl, 1 mM dithiothreitol (DTT), 1% Triton X-100). The cells were lysed by sonication (Sonosmasher, ULH-700s, microtip, 70% power for 200 CNT (10 s on, 20 s off) with ice cooling), and then centrifuged (1 h, 13,000× *g*). The supernatant was filtered (Millipore syringe filter, 0.45 μm) and applied to 5 mL glutathione-agarose 4B (Incospharm, Korea) affinity column. The 5 mL column was washed with 50 mL washing buffer 1 (1× PBS, 1 mM DTT, and 1% Triton X-100) and 50 mL of washing buffer 2 (50 mM Tris/Cl (pH 8.0) and 1 mM DTT). The protein was then eluted with 10 mL elution buffer (50 mM Tris/Cl (pH 8.0), 1 mM DTT, 0.1% Triton X-100, and 10 mM glutathione). The purest fractions determined by 10% SDS-PAGE were combined and ultrafiltrated with an Amicon stirred cell (YM-30). During the ultrafiltration, desalting and buffer exchange were accompanied for the next column containing 180 mL of Sephadex G-100 resin (Sigma), which was previously washed with buffer 3 (25 mM Tris–HCl (pH 7.5), and 0.3 M NaCl). After being applied to the Sephadex G-100 column, the protein sample (~5 mL) was eluted with the same buffer at a flow rate of 0.3 mL/min. The purest fractions determined by 10% SDS-PAGE were combined, and the pooled fractions were ultrafiltrated with the Amicon stirred cell (YM-30). The purified protein in 30% (v/v) glycerol was frozen at −80 °C for long-term storage.

**Figure 2:**
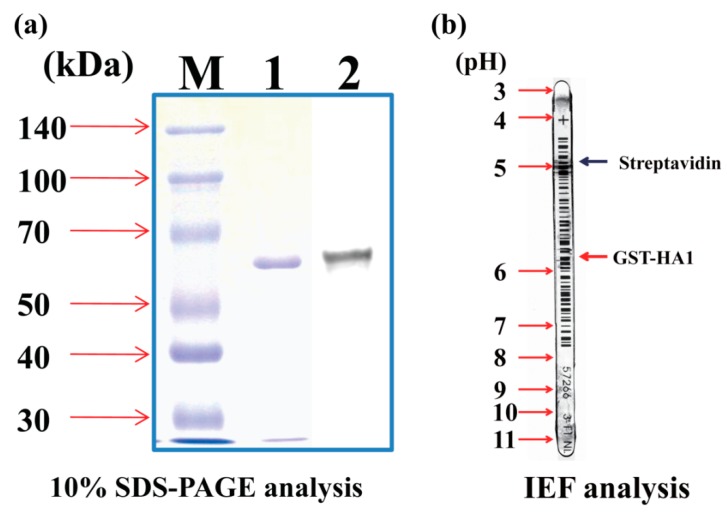
The (**a**) SDS-PAGE and (**b**) IEF analysis results from the purification of GST-tagged HA1.

The HA1-GST protein concentration was determined by the absorbance measurement at 280 nm in 8 M Urea (extinction coefficient: 96,720 M^−1^ cm^−1^) as well as by using a Bio-Rad protein assay system (Bio-Rad) with bovine serum albumin as the standard. The HA1-GST protein was observed in a single band of ~64 kDa by 10% SDS-PAGE after staining with Coomassie Blue and Western blot (Figure 2a). Lane 1 shows the purified HA1-GST, while lane 2 shows the Western blot result of the purified HA1-GST. The gel was stained with Coomassie brilliant blue. The Western blotting band was detected with GST–antibody–HRP.

The isoelectric point (pI) of the purified protein was confirmed by the isoelectric focusing (IEF) analysis with the control protein, streptavidin (Figure 2b). Streptavidin showed pI = ~5, while the purified HA1-GST showed pI = ~6 in the IEF analysis of the purified HA1-GST.

### 2.2. Fabrication and Electrical Characterization of the SiNW FETs

**Figure 3:**
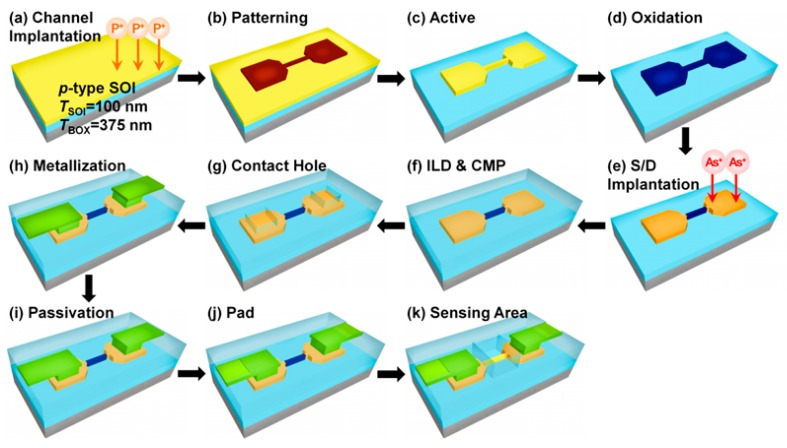
Schematic of the fabrication procedure for SiNW FET biosensors. A top-down approach was used to fabricate the SiNW FET-based influenza virus sensors.

The SiNW FETs were fabricated based on the top-down approach, which is advantageous compared to the bottom-up approach in terms of either the design of circuits and systems or the mass production because of its controllability and reproducibility, starting with boron doped at 4 × 10^15^ cm^−1^ 6 in. (100) silicon-on-insulator (SOI) wafers prepared through the implanted oxygen technique. The superficial 100 nm thick silicon was separated from the silicon substrate by 375 nm thick buried oxide (BOX). The SOI wafers were first dry-oxidized for 16 min at 950 °C to form 20 nm of buffer oxide as a protection layer for implantation. Consequently, the top Si layer thickness was thinned down to approximately 90 nm. Next, the channel implantation was conducted with phosphorus ion (energy: 40 keV; dose: 3 × 10^13^ cm^−2^) for the *n*-type accumulation mode device. After stripping the buffer oxide layer by the diluted HF solution, the annealing process was conducted at 950 °C for 30 min in a nitrogen environment (Figure 3a). The active region of the device was patterned on the top Si layer by the mix-and-match process composed of e-beam lithography for defining the nanowire (length *L* = 4 µm; width *W* = 70 nm) and conventional photolithography for forming large silicon features like source and drain (Figure 3b). The SiNW and the source/drain were anisotropically etched with the HBr/O_2_-based inductively coupled plasma by hydrogen silsesquioxane (HSQ) for e-beam lithography and a photoresist (PR) for photolithography as an etch mask (Figure 3c). A 10 nm thick oxide was thermally grown in a furnace for 42 min at 850 °C to form an implant slow down layer and reduce the size of the SiNW. An additional 10 nm thick oxide was then deposited for 14 min at 780 °C by means of a low-pressure chemical vapor deposition system (Figure 3d). Conventional photolithography was employed to form a photoresist mask that covered only the SiNW channel for the doping implantation of the source and drain (S/D) regions of the devices. The S/D regions of the *n*-type accumulation-mode SiNW FETs were doped with arsenic at 50 keV with a dose of 3 × 10^15^ cm^−c^. After the PR strip, the annealing step was followed at 1000 °C for 30 min in a nitrogen environment for the dopant activation (Figure 3e).

Serial processes for the back-end-of-line fabrication of the SiNW FET sensor, which are compatible with the conventional CMOS process technology, were performed. A 50 mm thick oxide was deposited as an interlayer dielectric via high-density plasma chemical vapor deposition, followed by the planarization process through chemical mechanical planarization (CMP) (Figure 3f). After the CMP process, the contact etching was performed using a combination of photolithography and magnetic enhanced reactive ion etching (MERIE) in CHF_3_/CF_4_ plasma (Figure 3g). The formations of the metal interconnection line and the Al electrode were afterwards performed step-by-step using Al sputtering, photolithography, and MERIE in BCl_2_/Cl_2_ plasma (Figure 3h). Tetraethyl orthosilicate (TEOS) was deposited as the passivation oxide to protect the metal line and prevent the leakage current between the ionic buffer solution and the metal line during the biosensor operation in an aqueous environment (Figure 3i). The oxide layer on the metal pad region was etched by photolithography and MERIE in CHF_3_/CF_4_ plasma to measure the electrical characteristics of the fabricated devices on the 6 in. SOI wafer (Figure 3j).

The sensing area on the SiNW parts was opened by removing the TEOS passivation oxide via MERIE in CHF_3_/CF_4_ plasma. The biomolecule analyte in the aqueous environment can be detected by means of contacting the SiNW surface with the analyte through the removal of the oxide layer on the SiNW part. Finally, the alloy process was conducted to remove the dangling bonds and complete the whole process (Figure 3k).

**Figure 4:**
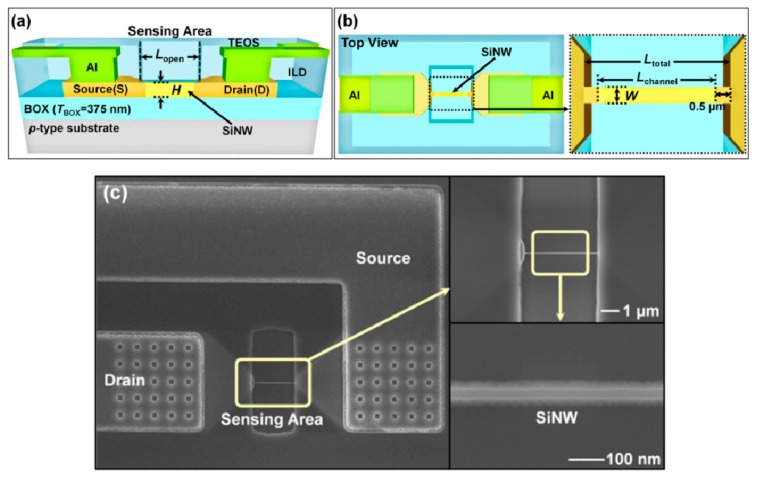
Schematics of (**a**) the cross section and (**b**) top view of SiNW FETs (not to scale). (**c**) SEM images of SiNW FET (*W* = 30 nm, *H* = 70 nm, *L*_open_ = 2 µm, and *L*_channel_ = 1 µm).

Figure 4 shows the schematic view (Figure 4a,b) and the scanning electron microscope (SEM) image (Figure 4c) of the fabricated SiNW FETs. The height, channel length, length of the open sensing area, and total length were *H* = 70 nm, *L*_open_ = 2 µm, and *L*_channel_ = 1 µm, respectively. *L*_channel_ depicts the separation between the S/D metallurgical junctions, and is effectively determined from *L*_open_ and two offsets of 0.5 μm (Figure 4b). The SiNW width *W* was varied from 30 to 400 nm through e-beam lithography. Figure 4c shows the case of *W* = 30 nm.

As shown in Figure S1a,b in Supplementary Materials S1, a total of 11 masks were used in the wafer-scale process, and 83 SiNW FETs were integrated in each chip. The sensing area was located at the center of each chip, while 258 pads were located on the edge of each chip. The controllability of the width and the alignment of the individual SiNWs was verified by the SEM images of the fabricated SiNW FETs (Figure S1c in Supplementary Materials S1). Noticeably, 99% of the integrated SiNWs on the 6 in. SOI wafer was built as the designed value. Therefore, the proposed top-down fabrication method is suitable for high-quality mass production, which is the definite and distinguished advantage of the top-down approach.

For the microfluidic channel measurement, a polydimethylsiloxane (PDMS) microfluidic channel (length = 4 mm, width = 2.5 mm, height = 200 µm) was constructed on a 9 mm × 9 mm chip for the fluidic transport of the sample solution. The construction procedure is described in Figure S2 in Supplementary Materials S2. The electrical characterization for the HA1-GST detection based on the SiNW FETs was performed using the measurement setup presented in Figure S3 in Supplementary Materials S2. The electrical measurement was conducted using an HP4156C (Keysight, Santa Rosa, CA, USA) semiconductor parameter analyzer, which controls the liquid gate voltage *V*_LG_, drain voltage *V*_D_, and source voltage *V*_S_ after the flow of the electrolyte solution has been sufficiently stabilized. 

### 2.3. Surface Functionalization of the SiNWs

For the HA1-GST detection with high sensitivity and selectivity, surface functionalization and immobilization were indispensable for the specific binding of HA1-GST to the SiNW surface in the SiNW FETs. The essence of our approach is to use CMP-NANA as a probe that specifically binds both to the aldehyde self-aligned monolayer (SAM) on the SiNWs and to HA1 simultaneously. We used two kinds of linkers (i.e., 3-aminopropyltriethoxysilane (APTES) and glutaraldehyde (GA)) and one probe (CMP-NANA) to bind the HA1-GST on the native oxide surface of the SiNW.

**Figure 5:**
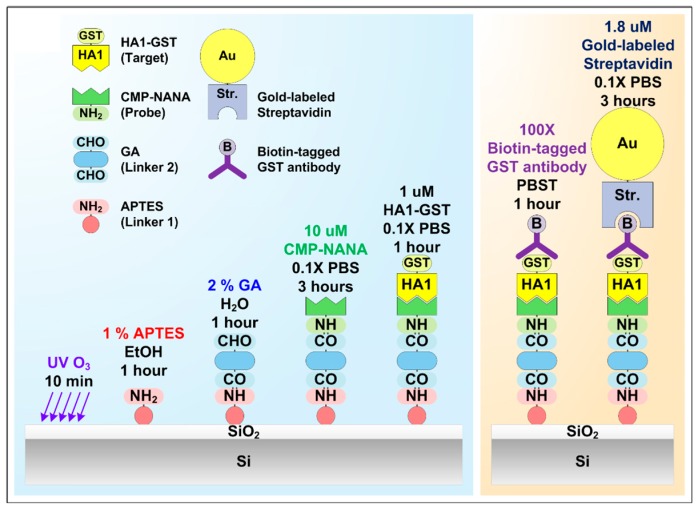
Scheme of the surface functionalization and immobilization of CMP-NANA and HA1 (left panel) and the preparation for tagging HA1 with gold nanoparticles for SEM analysis (right panel).

Figure 5 shows the scheme of the surface functionalization and immobilization of CMP-NANA and HA1 (left panel). First, after constructing the PDMS microfluidic channel, the device was treated by UV O_3_ for 600 s to introduce more hydroxyl (–OH) groups on the Si oxide surface, which is a well-known SAM method employing surface amine and aldehyde modification, and is for the specific binding of the amine group of CMP-NANA to SiNW [40]. APTES (first linker) and GA (second linker) were then sequentially applied and followed by the interaction with CMP-NANA. The device was functionalized by exposing the surface to 1% APTES in 95% ethanol for 1 h to form the SAM with the aldehyde group on the native oxide surface of the SiNWs. It was then washed with ethanol and dried with nitrogen and heated on a hot plate for 10 min at 120 °C. After the formation of the amine-terminated SAM by the preceding process, 2% GA in deionized water was coupled to the NH_2_ surface of the SiNW for 1 h, followed by a thorough washing with deionized water. GA was chosen as the second linker because it has two aldehyde groups in its top and bottom; therefore, it can bind individually to all the amine groups of APTES and CMP-NANA (left panel, Figure 5).

Subsequently, 10 µM CMP-NANA in 0.1× PBS was added to the CHO surface to employ the sialic acid moiety as a probe for HA1. CMP-NANA was chosen as a critical probe in our case because it has both the sialic acid moiety and the amine (–NH_2_) group. The former mimicked the process of a typical viral attachment to the cell surface that is activated by the binding of HA1 to the sialic acid group of the host cell surface. The latter provided the selected probe with the binding ability either to aldehyde (–CHO) or carboxylic acid (–COOH) that is widely available to the linker design for the specific binding of HA1 to the SiNW surface.

After excess material was removed by rinsing with 0.1× PBS, HA1-GST in 0.1× PBS was added to the surface for 40 min. The sample was diluted with the same 0.1× PBS to verify the HA1 detection in the femtomolar concentration range.

**Figure 6:**
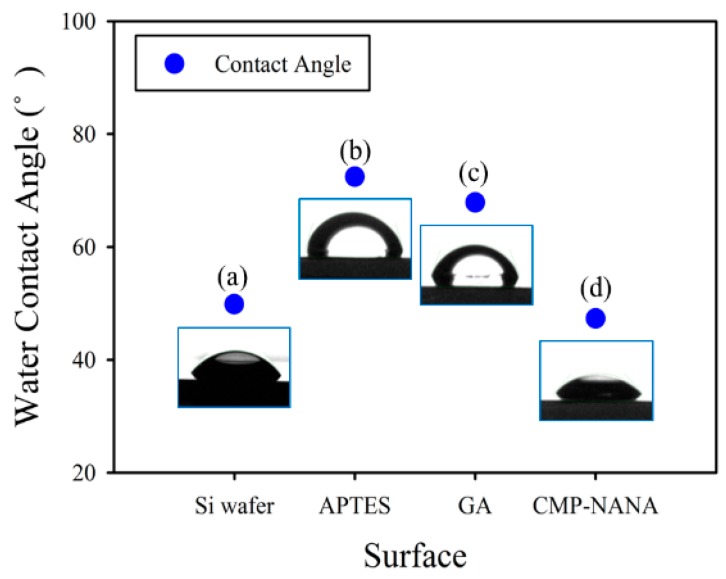
Water droplet contact angle measurements on four different functionalized Si wafer surfaces. (**a**) Bare Si wafer (49.8°), (**b**) APTES-functionalized surface (72.4°), (**c**) Glutaraldehyde-functionalized surface (67.8°), and (**d**) CMP-NANA immobilized surface (47.3°).

The contact angle of each step was also observed during the surface modification processes, and the well-modified surface was confirmed. The contact angle of the bare Si wafer with native oxide was 49.8° and changed into 72.4° (after APTES functionalization), 67.8° (after APTES/GA functionalization), and 47.3° (APTES/GA/CMP-NANA immobilization) (Figure 6). The changes in the contact angle indicated that the functional groups were adequately modified. We now finally confirmed the surface immobilization of CMP-NANA.

In addition, ELISA using GST–antibody–conjugated horseradish peroxidase (GST–antibody–HRP) was performed at each step of functionalization. The ELISA experiments were generally conducted to detect the protein–antibody interaction on the coated plate. We modified the conventional ELISA by performing on the Si wafer, by which we were able to determine whether or not the immobilized CMP-NANA on the surface binds to the HA1 domain. A 2 × 2 cm Si wafer was prepared, and CMP-NANA was immobilized on the Si wafer surface as described earlier. The immobilized surface was incubated with 5% (w/v) bovine serum albumin in 1× PBST (0.1% TWEEN-20 in PBS) solution for 1 h at room temperature to block the nonspecific binding, then rinsed with 1× PBST thrice. A 3 μM HA1-GST protein was added to the surface-immobilized CMP-NANA for 1 h at room temperature, then rinsed with 1× PBST thrice. The GST–antibody–HRP (1:1000 in PBST, Santa Cruz, USA) was added to the bound HA1-GST for 1 h at room temperature. After which, the wafer was rinsed with 1× PBST thrice and dried in N_2_ gas. The surface was then immersed in 2 mL *o*-phenylenediamine (OPD) solution to develop the color. Accordingly, 100 µL of the solution was removed every 5 min, and 100 µL of 2.5 N H_2_SO_4_ was added to terminate the reaction. The absorbance was measured at 492 nm using a TRIAD microplate reader (Dynex Technologies, VA, USA).

**Figure 7:**
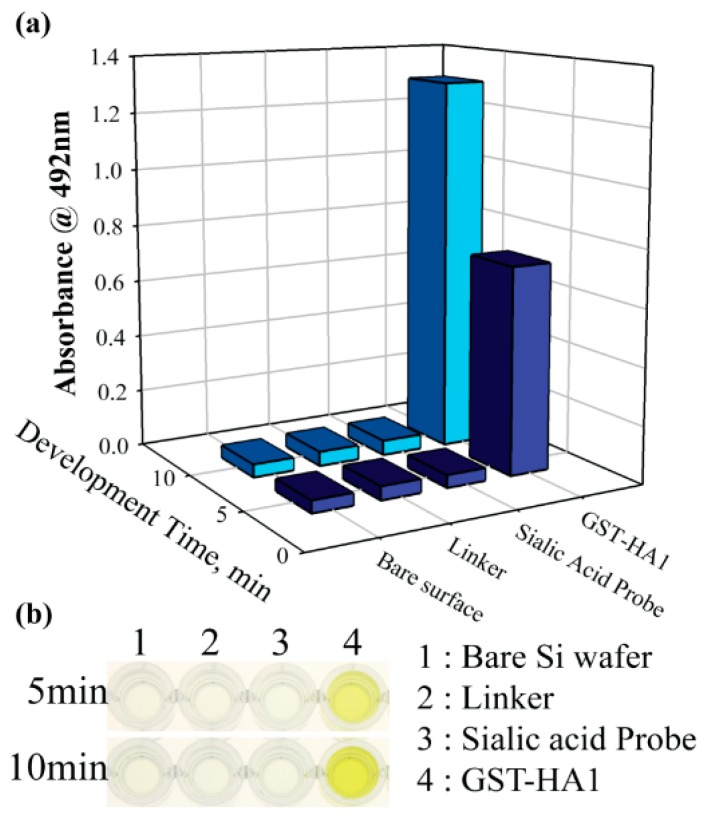
ELISA of functionalized Si wafer. (**a**) Absorbance was measured at 492 nm after sequential functionalization of Si wafer; bare surface, surface immobilized with linker (APTES and GA), surface functionalized with CMP-NANA, and addition of HA1 domain to the surface of functionalized CMP-NANA. (**b**) Color changes of terminated reaction with 2 N H_2_SO_4_ in 96-well plate.

Figure 7 clearly shows that the absorbance of the bare Si, linkers (after APTES/GA functionalization), and sialic acid probe (after APTES/GA/ CMP-NANA immobilization) was low (0.04–0.05) at a 492 nm wavelength, whereas the absorbance signal of the sample employing the GST–antibody–HRP after the APTES/GA/CMP-NANA/ HA1-GST immobilization was much higher (1.2–1.3) within 10 min. This result suggests that the HA1 specifically binds to the CMP-NANA immobilized on the SiNW surface. Thus, CMP-NANA and GA play the roles of a useful probe and an intermediate linker, respectively, for the specific binding of HA1 to the surface-modified SiNWs.

Furthermore, the SEM images were obtained to confirm that the change of the SiNW FET’s electrical characteristics came from the bound HA1-GST on the SiNW surface. The right panel in Figure 5 shows the functionalization sequences to obtain the SEM images. Subsequently, 100× biotin-tagged GST–antibody (Santa Cruz, CA, USA) was reacted with the surface bound HA1-GST in 1× PBST solution for 1 h at room temperature, then rinsed with 1× PBST and dried with nitrogen thrice after the binding of the HA1-GST to the SiNW surface by a microfluidic channel and a syringe pump. A 1.8 µM gold-labeled streptavidin (Sera care, Milford, MA,, USA) was reacted with the biotin bound to the surface for 3 h at room temperature, then rinsed with 0.1× PBS and dried with nitrogen thrice.

**Figure 8:**
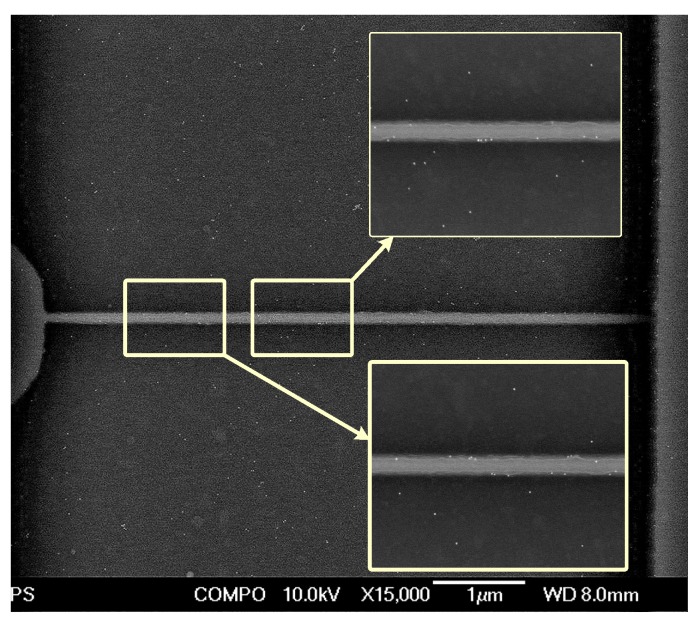
The SEM image of SiNW after the gold nanoparticle-tagging treatment to verify the immobilization of CMP-NANA and HA1.

Well-known strong bindings of the GST–GST antibody and biotin–streptavidin make it possible to confirm the successful immobilization of HA1 on SiNW by SEM analysis. Figure 8 shows the SEM image of SiNW after all treatments, in which many gold nanoparticles were observed to be located on and around SiNW. Thus, it was confirmed that the target protein HA1 was successfully immobilized on the SiNWs.

## 3. Result and Discussion

### 3.1. pH Sensing in the SiNW FET

The pH sensing was performed to confirm a fundamental operation of the fabricated SiNW FETs. The SiNW FET surface was functionalized using APTES to obtain a surface with an amine (–NH_2_). By using 0.1 M potassium phosphate buffers (pH 5–9) for the pH solutions, we eliminated the possible side effects caused by the alkali metal ions by maintaining a constant concentration of alkali metal ions. Therefore, our SiNWs could selectively respond to the presence of hydrogen ions.

**Figure 9:**
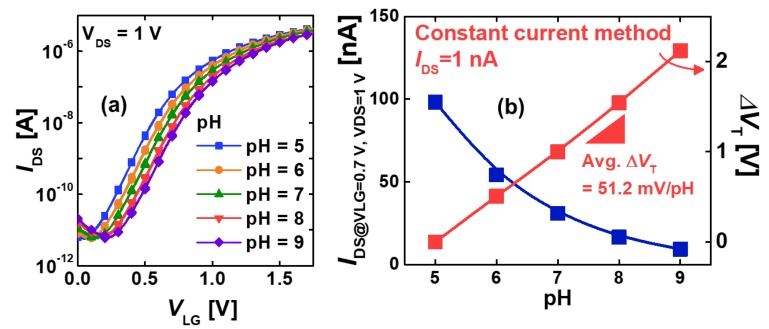
(**a**) Transfer characteristics of SiNW FETs with varying pH values. (**b**) the pH-dependencies of the drain-to-source current (*I*_DS_) and Δ*V*_T_ in SiNW FETs. The sensitivity of Δ*V*_T_ = 51.2 mV/pH was comparable to the Nernst limit.

Figure 9a shows the transfer characteristics of the n-type SiNW FETs with varying pH values, where *I*_DS_ denotes the drain-to-source current, and the drain-to-source voltage (*V*_DS_) is fixed at 1 V. The –NH_2_ group was protonated to –NH_3_ at a low pH, resulting in a greater positive potential. In contrast, the –SiOH group was deprotonated to –SiO– at a high pH, resulting in a greater negative potential. The surface potential at the SiO_2_/SiNW interface was confirmed to be well-modulated by the pH values. The average shift of the threshold voltage *V*_T_ (Δ*V*_T_) by pH was 51 mV/pH (Figure 9b), which is comparable to the ideal Nernst limit (∼59.2 mV/pH at 25 °C).

### 3.2. Electrical Detection of HA1-GST

The electrical detection of HA1-GST was conducted for the SiNW FETs after the APTES/GA/CMP-NANA/HA1-GST immobilization. The PBS solution was used as the background environment.

**Figure 10:**
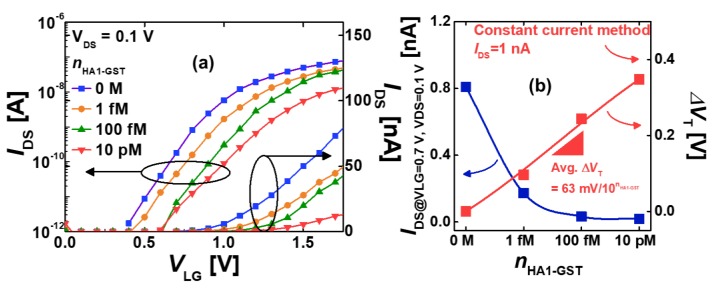
(**a**) Measured transfer curves of the SiNW FETs after APTES/GA/CMP-NANA/HA1-GST immobilization, depending on the concentration of HA1-GST. (**b**) Measured *I*_DS_ and Δ*V*_T_ with various concentrations of HA1-GST.

Figure 10a shows the measured transfer curves of the SiNWs with *W* = 70 nm at *V*_DS_ = 0.1 V depending on the HA1-GST concentration (*n*_HA1-GST_). The case of 0 M suggests the condition that only the PBS solution was flowing through the microfluidic channel. The transfer curve was clearly shifted to a positive *V*_LG_ direction with the increase of the HA1-GST concentration. The observed shift was very consistent with the pI value ~6 of HA1-GST (Figure 2b) considering the pH value = 7.4 of the PBS solution because the used SiNW operated in the *n*-type accumulation-mode FET. SiNW effectively felt a larger amount of negative charges and depleted more and more electrons as the HA1-GST concentration increased, resulting in the current decreasing and the FET threshold voltage increasing to a positive *V*_LG_ direction.

The voltage–concentration plot in Figure 10b is highly linear, and the HA1-GST sensitivity was determined as Δ*V*_T_ = 63 mV/dec. This reveals an ultralow detectable range (1 fM) of the target protein HA1 by the fabricated SiNWs. Therefore, the CMP-NANA is verified to be a potentially useful probe for the virus protein and can play the role of ultrasensitive virus detection with the silica-binding surface (i.e., Si or SiO_2_) by combining APTES and GA linkers.

The previous Si FET-based HA detection [41] achieved Δ*V*_T_ < 100 mV in the HA concentration range 10^−18^–10^−8^ M, and Δ*V*_T_ = ~20 mV at 1 fM HA. In this study, we achieved Δ*V*_T_ = 112 mV at 1 fM HA (Figure 10b). The noticeably higher sensitivity in our work is attributed to the combined use of CMP-NANA and SiNWs (high surface-to-volume ratio) and confirms the usefulness and appropriateness of immobilizing the SiNWs with CMP-NANA for the FET-based electrical detection of HA. 

In addition, the signal-to-noise ratio (SNR) of our SiNW FETs, defined as SNR = Δ*I* / δ*I*, was evaluated as 3433 (where Δ*I* = 35 pA and δ*I* = 103 nA denote the measured current-signal response and the root-mean-squared current noise amplitude, respectively). Herein, Δ*I* was determined as the current change from pH 10 to pH 4, and δ*I* was obtained by integrating the current noise power spectral density (S_ID_) from 10 Hz to 1 kHz and square-rooting the result. The S_ID_ was taken from the low-frequency noise characteristics of our SiNW FETs. The observed SNR is comparable to [42] and will be improved and comprehensively characterized in further study.

## 4. Conclusions

In summary, we demonstrated herein the ultrasensitive electrical detection of the HA1 domain of HA, which is critically involved in influenza virus infection, by combining SiNW FETs and the functionalization scheme based on the CMP-NANA probe, GA, and APTES linkers. To the best of our knowledge, we provide the first demonstration of detecting not the specific nucleic acid sequences inside the influenza virus particles, but the viral surface protein on the influenza virus itself, by the top-down SiNW FET approach. Even femtomolar concentrations of HA1 can be detected (Δ*V*_T_ = 63 mV/dec, Δ*V*_T_ = 112 mV at 1 fM HA1) by virtue of the stable and efficient CMP-NANA probe and intermediate GA linker, and the high surface-to-volume ratio of the SiNWs. Furthermore, a good linearity and reasonable SNR were verified. The mass-producible functionalized SiNW-based sensor array can potentially realize fast, label-free, and exact POC yes/no diagnosis of the influenza virus.

## Supplementary Materials

The following are available online at https://www.mdpi.com/1424-8220/19/20/4502/s1, Controllability of top-down processed SiNWs on the 6 in SOI wafer (S1) and Microfluidic Channel and Electrical Characterization of the SiNW FETs (S2).
