# Supplementary material for: Ultrasensitive Electrical Detection of Hemagglutinin for Point-of-Care Detection of Influenza Virus Based on a CMP-NANA Probe and Top-Down Processed Silicon Nanowire Field-Effect Transistors
Source: Sensors (Basel). 2019 Oct 17;19(20):4502. doi: 10.3390/s19204502 (PMC6832293; doi:10.3390/s19204502)
Supplement: Supplementary file 1 [file sensors-19-04502-s001.pdf]

sensors

Supplementary Materials

Ultrasensitive Electrical Detection of Hemagglutinin for Point-of-care of Influenza Virus based on CMP-NANA Probe and Top–down Processed Silicon Nanowire Field-effect Transistors

Mihee Uhm,[1] Jin-Moo Lee,[2] Jieun Lee,[1] Jung Han Lee,[3] Sungju Choi,[1] Byung-Gook Park,[3] Dong Myong Kim,[1] Sung-Jin Choi,[1] Hyun-Sun Mo,[1] Yong-Joo Jeong,[2,*] and Dae Hwan Kim[1,*]

1 School of Electrical Engineering, Kookmin University, Seoul 02707, Korea; ljss88@naver.com (M.U.); taiji11jieun@kookmin.ac.kr (J.L.); sungjuchoi@kookmin.ac.kr (S.C.) dmkim@kookmin.ac.kr (D.M.K.); sjchoiee@kookmin.ac.kr (S.-J.C.); tyche@kookmin.ac.kr (H.-S.M.); drlife@kookmin.ac.kr (D.H.K.)
2 School of Applied Chemistry, Kookmin University, Seoul 02707, Korea; elzem@kookmin.ac.kr (J.-M.L); jeongyj@kookmin.ac.kr (Y.-J.J.)
3 School of Electrical and Computer Engineering, University of Seoul, Seoul 02504, Korea; kusa159@snu.ac.kr (J.H.L); bgpark@snu.ac.kr (B.-G.P.)
* Correspondence: jeongyj@kookmin.ac.kr (Y.-J.J.); drlife@kookmin.ac.kr (D.H.K.)

S1. Controllability of top-down processed SiNWs on the 6 in SOI wafer

In this study, SiNW FETs were integrated on 6 in. SOI wafer. Figures S1(a) and (b) depict the fabricated 6 in. SOI wafer and its 9 mm × 9 mm single chip, respectively. The controllability of the width and the alignment of the individual SiNWs was verified by the SEM images of the fabricated SiNW FETs [Fig. S1(c)]. 99% of the integrated SiNWs on the 6 in. SOI wafer was found to be built as the designed value.

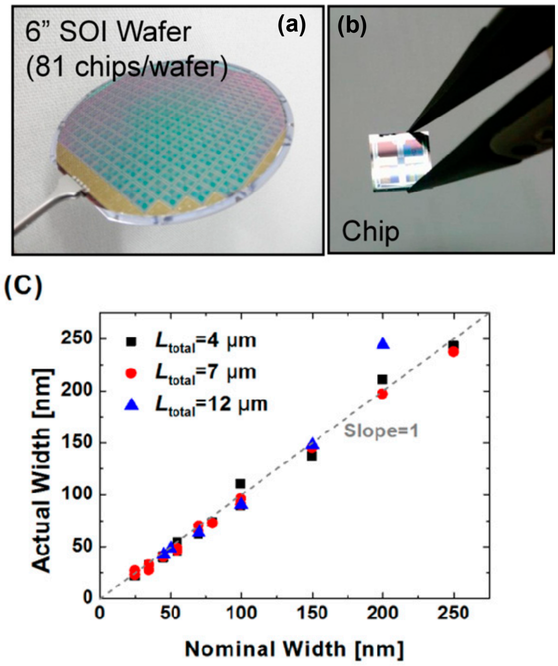

Figure S1. (a) Optical image of the fabricated 6-inch SOI wafer, (b) its single chip (9 mm × 9 mm). (c) Plot of the nominal *W* versus actual *W* of SiNWs integrated on the 6-inch SOI wafer. The controllability of the width and alignment of SiNWs are verified by the SEM images of the fabricated SiNW FETs. It shows that

over 99 % of SiNWs among all integrated ones on the 6-inch SOI wafer have been built as the designed value.

S2. Microfluidic Channel and Electrical Characterization of the SiNW FETs

A polydimethylsiloxane (PDMS) microfluidic channel (length = 4 mm, width = 2.5 mm, height = 200 μm) was constructed on a 9 mm × 9 mm chip for the fluidic transport of the sample solution (Fig. S2). First, the PDMS master was fabricated on a 1 mm-thick 4 in. Si wafer [Fig. S2(a)]. The master was filled with a fully stirred mixture of 40 g Sylgard-184 silicone elastomer and 4 g Sylgard-184 silicon elastomer curing agent. It was then baked at 80 °C for 3 h. The cured PDMS was taken off and cut to fit with the final chip dimensions around the channel. The PDMS microfluidic channel was attached on the chip by exposing it UV O_3 for 120 s before being bonded together [Figs. S2(b) and (c)]. It was then heated on a hot plate at 120 °C for 10 min. Figure S2(d) shows the final measurement setup with tubing lines (inner diameter = 500 μm, outer diameter = 1.6 mm) on a probe station.

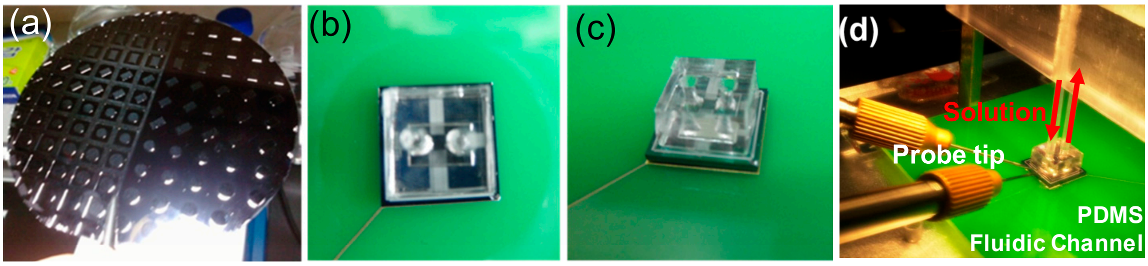

Figure S2. Polydiemthysiloxane (PDMS) microfluidic channel. PDMS microfluidic channel was installed on the chip (9 mm × 9 mm) for fluidic transport of the sample solution. (a) The fabricated PDMS microfluidic channel master on 4-inch Si wafer. (b) Top view and (c) bird's eye view of the biosensor chip bound with PDMS microfluidic channel. (d) Measurement setup with tubing lines (inner diameter = 500 μm, outer diameter = 1.6 mm) on the probe station. The arrows display the flow direction of sample solution.

Figure S3 shows a schematic diagram of the measurement setup using the PDMS microfluidic channel and the syringe pump. The arrows represent the flow direction of the used sample, and the solutions were supplied to the SiNW sensor at a constant and typical flow velocity (1.7 mm/s) controlled by the syringe pump [1]. Liquid gate biases were applied with the Ag/AgCl (3M KCl) reference electrode (eDAQ, Australia) in a beaker to control the reference electrical potential of the electrolyte solution [2]. The electrical measurement was conducted using the semiconductor parameter analyzer (Agilent 4156C, USA) with the liquid gate voltage V_{LG}, drain voltage V_D, and source voltage V_S after the flow of the electrolyte solution has been sufficiently stabilized.

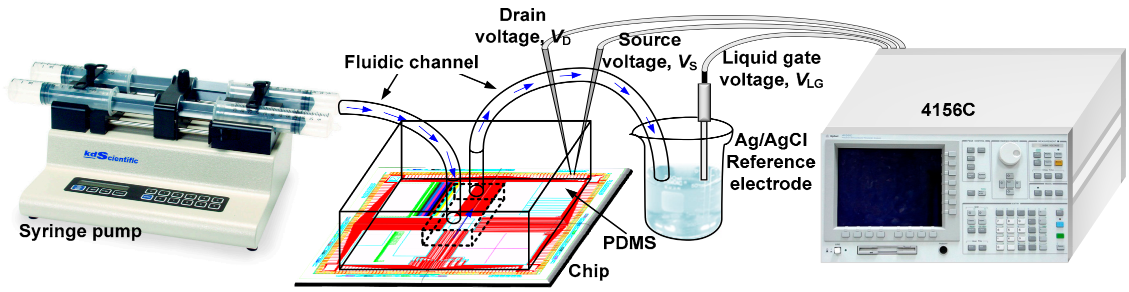

Figure S3. Schematic diagram illustrating the measurement setup. Sample solutions were supplied to the SiNW by syringe pump along the arrows through microfluidic channel, finally reach the beaker.

References

1. Lin, T.; Wang, G.; Li, A.; Zhang, Q.; Wu, C.; Zhang, R.; Cai, Q.; Song, W.; Yuen, K.-Y. The hemagglutinin structure of an avian H1N1 influenza A virus. *Virology* **2009**, 392, 73–81.
2. Zaitsev, V.; von Itzstein, M.; Groves, D.; Kiefel, M.; Takimoto, T.; Portner, A.; Taylor, G. Second Sialic Acid Binding Site in Newcastle Disease Virus Hemagglutinin-Neuraminidase: Implications for Fusion. *J. Virol.* **2004**, 78, 3733–3741.

© 2019 by the authors. Licensee MDPI, Basel, Switzerland. This article is an open access article distributed under the terms and conditions of the Creative Commons Attribution (CC BY) license (http://creativecommons.org/licenses/by/4.0/).
